# Labelled regulatory elements are pervasive features of the macrophage genome and are dynamically utilized by classical and alternative polarization signals

**DOI:** 10.1093/nar/gkz118

**Published:** 2019-02-25

**Authors:** Attila Horvath, Bence Daniel, Lajos Szeles, Ixchelt Cuaranta-Monroy, Zsolt Czimmerer, Lilla Ozgyin, Laszlo Steiner, Mate Kiss, Zoltan Simandi, Szilard Poliska, Nikolas Giannakis, Emanuele Raineri, Ivo G Gut, Benedek Nagy, Laszlo Nagy

**Affiliations:** 1 Department of Biochemistry and Molecular Biology, Faculty of Medicine, University of Debrecen, H-4032 Debrecen, Hungary; 2 Johns Hopkins University School of Medicine, Department of Medicine and Biological Chemistry, Institute for Fundamental Biomedical Research, Johns Hopkins All Children’s Hospital, Saint Petersburg, FL 33701, USA; 3 UD-GenoMed Medical Genomic Technologies Ltd., Nagyerdei krt. 98., H-4032 Debrecen, Hungary; 4 Centro Nacional de Analisis Genomico (CNAG-CRG), Center for Genomic Regulation (CRG), Barcelona Institute for Science and Technology (BIST), C/Baldiri Reixac 4, 08028 Barcelona, Spain; 5 Universitat Pompeu Fabra (UPF), Plaça de la Mercè 10, 08002, Barcelona, Spain; 6 Department of Mathematics, Eastern Mediterranean University, Famagusta, North Cyprus, Mersin 10, Turkey

## Abstract

The concept of tissue-specific gene expression posits that lineage-determining transcription factors (LDTFs) determine the open chromatin profile of a cell via collaborative binding, providing molecular beacons to signal-dependent transcription factors (SDTFs). However, the guiding principles of LDTF binding, chromatin accessibility and enhancer activity have not yet been systematically evaluated. We sought to study these features of the macrophage genome by the combination of experimental (ChIP-seq, ATAC-seq and GRO-seq) and computational approaches. We show that Random Forest and Support Vector Regression machine learning methods can accurately predict chromatin accessibility using the binding patterns of the LDTF PU.1 and four other key TFs of macrophages (IRF8, JUNB, CEBPA and RUNX1). Any of these TFs alone were not sufficient to predict open chromatin, indicating that TF binding is widespread at closed or weakly opened chromatin regions. Analysis of the PU.1 cistrome revealed that two-thirds of PU.1 binding occurs at low accessible chromatin. We termed these sites labelled regulatory elements (LREs), which may represent a dormant state of a future enhancer and contribute to macrophage cellular plasticity. Collectively, our work demonstrates the existence of LREs occupied by various key TFs, regulating specific gene expression programs triggered by divergent macrophage polarizing stimuli.

## INTRODUCTION

Specification of cellular identity and function are controlled by lineage-determining transcription factors (LDTFs) (1). Many LDTFs has been proposed to act as pioneer factors that have the intrinsic ability to bind their recognition sequences within condensed chromatin, making these genomic loci accessible to other transcription factors (TFs) (2). In macrophages, the master regulator of myeloid differentiation, PU.1 (also known as SPI1), is an essential LDTF and a potential pioneer factor, which is responsible for enhancer selection upon differentiation (3). It has been suggested that enhancers established and marked by PU.1 provide additional options for the cells to respond to environmental cues (4). However, recent studies in macrophages and B cells suggest a distinct model in which PU.1, and a relatively small set of additional LDTFs, act collaboratively to bind chromatin in a cell type-specific manner. These collaborative interactions set the stage for signal-dependent transcription factors (SDTFs) to initiate their genomic programs (5,6).

Further investigations of such collaborative binding, using two mouse strains and an elegant experimental design utilizing naturally occurring genetic variations between the strains, revealed that the two macrophage LDTFs, PU.1 and CCAAT/enhancer-binding protein α (CEBPA), greatly impact each other’s binding. More specifically, mutations in the binding motif of PU.1 hindered the binding of CEBPA and vice versa. Importantly, although the binding of the SDTF nuclear factor-κB (NF-κB) was strongly dependent on the presence of an intact PU.1 and CEBPA motif, mutations in the binding motif of NF-κB had only marginal or no effect on the binding of PU.1 and CEBPA. The above findings provide evidence that LDTFs select enhancer elements by binding to variably spaced DNA-binding motifs in a collaborative manner (7).

Although much has been learned about the nature of collaborative functions, the binding properties of the main macrophage-specific TFs and their associations with open chromatin have not yet been thoroughly examined. Nevertheless, several studies have described the enrichment of PU.1, CEBP, Interferon regulatory factor (IRF), Runt-related transcription factor (RUNX) and Activator protein 1 (AP-1) motifs at macrophage-specific regulatory regions (7–9). Among these TFs, PU.1 is known as an inducer of myeloid differentiation (10), CEBPA/B and PU.1 mediate the trans-differentiation of fibroblasts into macrophage-like cells (11); RUNX1 is indispensable for the development of the hematopoietic system (12); IRF8 is essential for murine monocyte development and is known to have a distinct role in controlling inflammatory stimulus-inducible genes upon classical polarization (6,13) and finally, the AP-1 family member JUNB is required for proper macrophage polarization via both classical and alternative pathways (14). In line with that, recent works suggest that different TF modules provide gene regulatory plasticity to the cells when different activation programs are initiated by single, synergistic or opposing external signals (15–17). Nonetheless, SDTFs are also able to trigger unique transcriptional responses at genomic regions devoid of PU.1 and establish latent or *de novo* enhancers in classically and alternatively polarized macrophages (5,18).

These results raise the intriguing question whether the solo binding patterns of PU.1 and other TFs playing critical roles in macrophage biology are sufficient to determine chromatin openness and/or activity, and whether they have a deterministic role in establishing SDTF binding upon external stimuli. It has been shown that machine learning methods such as Random Forest or Support Vector Machines can be effectively used to identify and prioritize the most important features of the chromatin environment (histone marks, motif sequences, etc.) that affect TF binding (19–21) and enhancer activity (22). However, determinants of chromatin openness received little attention in these studies. Mapping of TF binding and accessible chromatin combined with computational approaches hold promise to determine the relationship between TF binding and chromatin openness. By using machine learning methods, we systematically assessed predictive models on chromatin openness using the binding patterns of LDTFs and other key TFs of macrophages (PU.1 CEBPA, IRF8, RUNX1 and JUNB). This modelling approach not only provides prediction accuracies, but also defines the relative importance of the studied TFs in predicting chromatin openness all over the macrophage genome.

Here we show that machine learning approaches are able to predict open chromatin from solely the cistromes of the studied TFs. This analysis not only predicted chromatin openness, but also pinpointed to the existence of a novel regulatory element class representing very large portions of the studied cistromes. These genomic regions have a unique feature of TF binding to low accessible or closed chromatin, which we termed labelled regulatory elements (LREs). Most of the key TFs of the macrophages are able to form LREs, and polarization signals can dynamically transform these sites to active enhancers in a stimulus-specific manner. Collectively, these findings expand and refine our understanding about TF binding and its relation to chromatin openness, revealing a novel class of regulatory elements, which most likely represents a critical state of enhancer activation and likely contributes to dynamic and graded gene expression regulation.

## MATERIALS AND METHODS

### Differentiation of bone marrow-derived macrophages

Isolation and differentiation were completed as described earlier (23). Bone marrow-derived cells isolated from isogenic C57BL6/J mice were differentiated for 6 days in the presence of L929 supernatant. At the 6th day of differentiation, cells were exposed to IL-4 (Interleukin-4, 20 ng/ml) and LPS (lipopolysaccharide, 100 ng/ml) for the indicated periods.

### ChIP

ChIP was performed as previously described (24–26) with minor modifications. The following antibodies were used: PU.1 (sc-352), IRF8 (sc-6058x), JUNB (sc-46x), CEBPA (sc-61x), RUNX1 (sc-8563x), STAT6 (sc-981x), p65 (sc-372), H4ac (millipore 06-866), H3K4me1 (ab8895), H3K27ac (ab4729) and RNAPII-pS2 (ab5095). Libraries were prepared by Ovation Ultralow Library Systems (Nugen) from two biological replicates according to the manufacturer’s instructions.

### GRO-seq

GRO-seq and library preparation were performed as described earlier (9). Libraries were generated from two biological replicates from untreated BMDMs.

### ATAC-seq

ATAC-seq was carried out as described earlier with minor modifications (27). Cells were scraped and counted to achieve 50 k/ml in ice-cold phosphate-buffered saline. Cell suspension was further diluted to 25 k/ml and nuclei were isolated with ATAC-LB (10 mM Tris–HCl pH 7.4, 10 mM NaCl, 3 mM MgCl_2_, 0.1% IGEPAL). Nuclei from 25 k cells from two biological replicates were used for tagmentation using Nextera DNA Library Preparation Kit (Illumina). After tagmentation, DNA was purified with Minelute PCR Purification Kit (Qiagen). Tagmented DNA was amplified with Kapa Hifi Hot Start Kit (Kapa Biosystems) using 16 polymerase chain reaction (PCR) cycles. Amplified libraries were purified again with Minelute PCR Purification Kit. The fragment size distribution of libraries was assessed with Agilent Bioanalyzer and sequenced on a HiSeq 2500 platform.

### ChIP-seq, GRO-seq and ATAC-seq analyses

Primary analysis of the ChIP-seq, GRO-seq and ATAC-seq raw reads was carried out using an analysis command line pipeline (28). Briefly, Burrows-Wheeler Alignment Tool (29) was used to align the reads to the mm10 genome assembly (GRCm38) with default parameters. MACS2 2.0.10 was used for predicting ATAC-seq and TF peaks (*q*-value ≤ 0.01). Artefacts were removed using the ENCODE blacklist (30). ‘Intergenic’ and ‘Intron’ regions were considered as distal elements from HOMER (v4.2) annotation. Reads per kilobase per million mapped reads (RPKM) values of the predicted peaks were calculated using BedTools coverageBed and bash scripts. DiffBind v2.8.0 (31) was used to infer differential binding sites from duplicates of STAT6 and p65 from CTR, 1 h IL-4 and 1 h LPS treated cells (*P*-value < 0.05), respectively, and from RNAPII-pS2 ChIP-seq time course experiments (*P*-value < 0.05 and FC > 2) measured on distal regions (normalized DiffBind occupancy >30) and on gene bodies (normalized DiffBind occupancy >50), using untreated samples as controls. K-means clustering of RNAPII-pS2 regions both on distal elements and gene bodies (mm10 RefSeq) was performed using kmeans function from the R package stats. GO analyses were performed using the clusterProfiler R package. Intersections, subtractions and merging of the predicted peaks were done with BedTools (v2.23.0). Regions were considered to be overlapped if there was at least one common nucleotide. Consensus sets were defined by merging overlapping regions (in at least 2 samples). Proportional Venn diagrams were generated with VennMaster. Genome coverage files (BedGraphs) for visualization purposes were generated by makeUCSCfile.pl, and then converted into tdf files using igvtools (IGV2.3, Broad Institute) with the ‘toTDF’ option. Genomic distribution was analysed using HOMER categories provided by annotatePeaks.pl (UTR regions were merged). *De novo* motif discovery was performed in the 150 bp vicinity of the peak summits using findMotifsGenome.pl with options ‘-length –len ‘12,14,16,18,20,22’ and ‘-size 200’ on the repeat-masked mouse genome (mm10r) from HOMER. Integrative Genomics Viewer (IGV2.3, Broad Institute) was used for data browsing (32) and creating representative snapshots. Normalized tag counts for Meta histograms and read distribution heat maps (RD plots) were generated by annotatePeaks.pl with ‘-ghist’ and ‘-hist 25’ options from HOMER on one representative example of duplicates and then visualized by R (ggplot2) or Java TreeView. Motif matrices were remapped using annotatePeaks,pl with the ‘-mscore’ option. Summits used for centring RD plots and motif remapping were identical to the summit of the peak with the highest MACS score from those used for deriving the consensus region. For a detailed list of NGS data, see Supplementary Table S5.

### Machine learning

Machine learning analyses were performed in R using the packages randomForest, e1071 and custom scripts. For training sets, 1000 sites were randomly chosen from both labelled and ‘HighAcc’ categories for Random Forest and Support Vector Machine models (repeat-masked mouse genome mm10r). To avoid the well-known issue of ‘overfitting’ in data mining, all models were built using a *k*-fold (*k* = 10) cross validation. In total, 31 Random Forest models were generated from all possible TF combinations (one TF only, *n* = 5; two TFs, *n* = 10; three TFs, *n* = 10; four TFs, *n* = 5; all five TFs - ‘full model’, *n* = 1). For validation sets, another 1000 sites were randomly chosen from both labelled and ‘HighAcc’ categories that were not used for learning processes. Contribution scores (MeanDecreaseGini) were calculated using randomForest function with the ‘importance = T’ option. Sensitivity, Specificity and ROC values were calculated with the caret and ROCR packages. Boxplot of the 31 models and the ROC plot were generated using ggplot2 R package. For SVM model, Pearson correlation coefficient was calculated on an independent validation set using stats packages in R.

### RT-qPCR

Total RNA was reverse transcribed with High-Capacity cDNA Reverse Transcription Kit (Applied Biosystems) according to the manufacturer’s protocol. Transcript quantification was performed by qPCR using SYBR green master mix (BioRad). Transcript levels were normalized to *Ppia*. Primers are available upon request.

## RESULTS

### Machine-learning approach points to the existence of TF-labelled, low accessible regulatory elements in macrophages

Although the genomic binding sites of PU.1 and other TFs in mouse macrophages have been characterized in detail (4,5,33–35), their relationship to chromatin openness have not yet been comprehensively studied and therefore it is not known whether their binding shows a strong correlation with chromatin openness or they can also bind to low accessible regions as well. In order to provide a baseline for our study, we mapped the accessible chromatin profile of bone marrow-derived macrophages (BMDMs). First, we performed Assay for Transposase Accessible Chromatin coupled with sequencing (ATAC-seq) (Supplementary Figure S1A) and found 45 253 accessible regions. To assess the sensitivity and specificity of our ATAC-seq approach, we compared our data to publicly available datasets generated in untreated BMDMs using DNase-seq (36) and FAIRE-seq (5). ATAC-seq detected more than twice as many accessible sites as DNase-seq or FAIRE-seq (Supplementary Figure S1B). Furthermore, ATAC-seq alone was able to identify 83% of all regions predicted by at least one method of the three; therefore, this technique proved to have the highest sensitivity to detect chromatin accessibility (lowest false negative rates). *De novo* motif analysis of the promoter-distal subset of open chromatin regions (32 561 regions) identified predominantly the PU.1 (ETS) motif (37.5%), TRE elements (28.4%) associated with certain members of the AP-1 family (37), CTCF (6.4%), CEBP (31.9%) and RUNX (12.4%) motifs, as well as PU.1-IRF composite elements called EICE (4.5%), indicating the important roles for the respective TFs in open chromatin organization (Supplementary Figure S1C).

**Figure 1.**
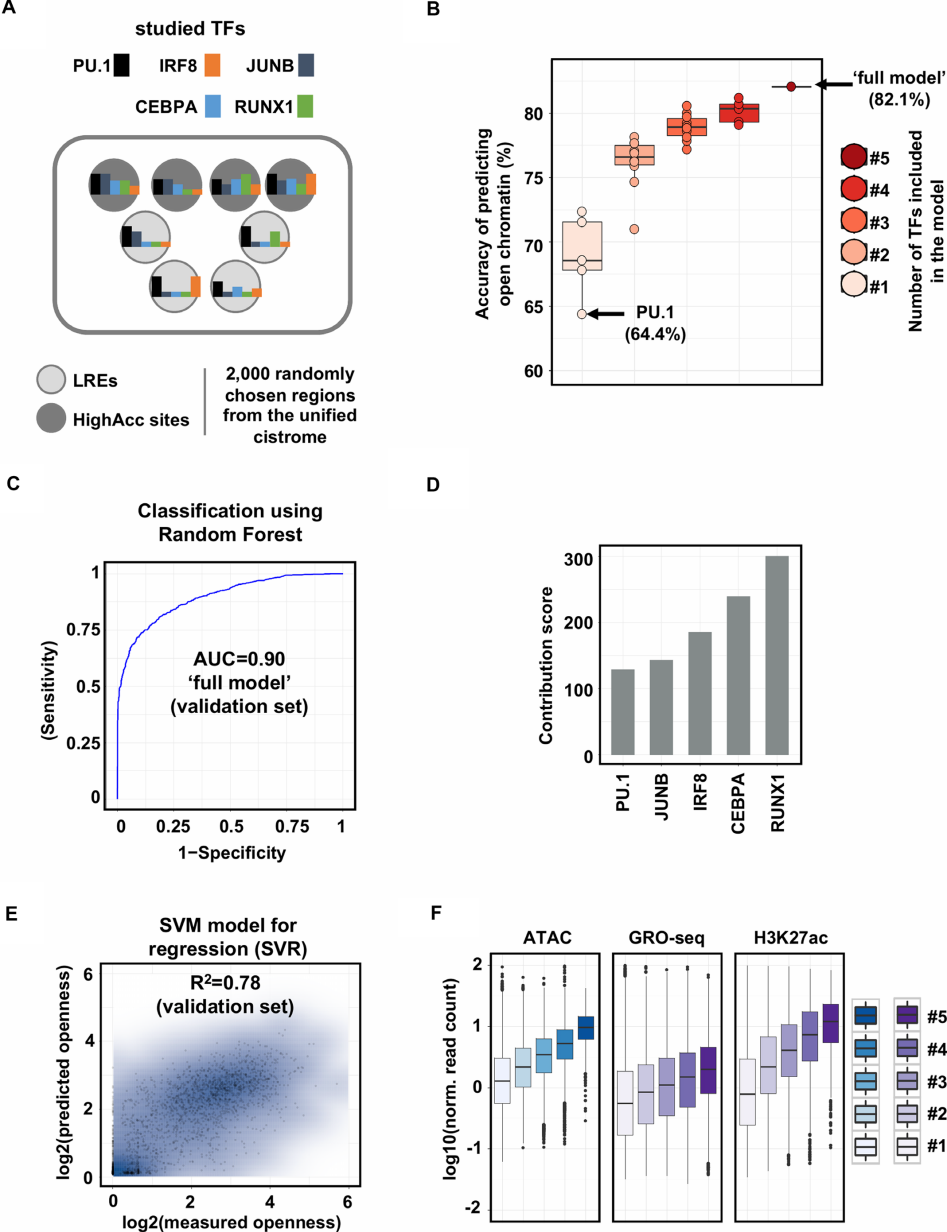
Machine-learning approach points to the existence of TF-labelled, low accessible regulatory elements in macrophages. (**A**) Flow chart of the machine learning approach. Labelled regulatory elements (LREs) are defined as one or more transcription factors (TFs) bound to low accessible chromatin. Highly accessible sites (HighAcc sites) are bound by one or more TFs and exhibit high ATAC-seq signals. (**B**) Box plots showing the accuracies of models in predicting open chromatin regions built with one TF (*n* = 5), two (*n* = 10), three (*n* = 10), four (*n* = 5) or five (*n* = 1, ‘full model’) TF(s). (**C**) ROC (receiver operating characteristic) curve of ‘full model’ (false positive rate and true positive rate are plotted on the *x*-axis and *y*-axis, respectively). AUC (area under the ROC curve) calculated on the validation set is also shown. (**D**) Bar plot showing the relative importance of PU.1 and the other four TFs assessed in the ‘full model’. (**E**) Scatter plot showing the Pearson correlation coefficient of predicted and measured openness calculated on the validation set. (**F**) Box plot representation of ATAC-seq, GRO-seq and H3K27ac signals on TF co-bound regions. #1-5 labels indicate the number of co-bound TFs present at the given genomic loci.

Next, we asked the question whether the binding of PU1, as one of the most prominent TFs in macrophages, has a deterministic role in chromatin openness or other TFs also have a strong contribution. To address this question, we decided to take computational approaches and studied the relative importance and predictive power of the binding of the key macrophage-specific TFs in the establishment of open chromatin. First, we carried out ChIP-seq experiments for the TFs whose motifs were enriched in our *de novo* motif analyses (PU.1, IRF8, JUNB, CEBPA and RUNX1) and had been reported to have important roles in macrophages as described above. Having predicted the genomic binding sites of each TFs, we merged these regions to create a unified set of genomic regions (i.e. regions bound by at least one of the studied TFs, Supplementary Table S1). We randomly selected 1000 regions from the unified set that did not overlap with predicted open chromatin, termed ‘labelled’ regulatory elements (LREs), and 1000 regions that overlapped with highly accessible regions (termed ‘HighAcc’ sites) (Figure 1A). Using Random Forest method, we systematically evaluated all possible combinations of TFs as input variables (for more details see ‘Materials and methods’ section) and measured the predictive power of these models. Interestingly, the model in which only PU.1 occupancy values were used resulted in a weak prediction accuracy of openness (64.4%), only slightly better than expected by chance. In addition, all the other TFs alone showed slightly better, but similarly weak predictive power. The systematic analysis of TF contributions showed that the more TFs were included in the model, the higher the prediction accuracy became (Figure 1B). The ‘full model’, in which all the TFs were included, predicted chromatin openness with high confidence on an independent validation set (Accuracy = 0.82, Sensitivity = 0.88, Specificity = 0.77, AUC = 0.90, Figure 1B and C; Supplementary Figure S1D).

The Random Forest method applied not only provides prediction accuracy, but also reports the relative importance of the features (TF binding) in predicting chromatin openness. This analysis showed that the majority of TFs preceded PU.1 in order based on their predictive power in the context of defining open chromatin regions (Figure 1D). Moreover, using the Support Vector Regressor method, we could predict chromatin openness not only qualitatively (classification task) but quantitatively as well with a high correlation coefficient between the measured and predicted ATAC-seq signal (Figure 1E). Finally, in line with our previous observation, we sought to reveal whether and how accessibility and activation of enhancers depend on the number of co-bound TFs. Having created disjunct subsets of the unified cistromes based on the number of co-bound TFs, we found that the more TFs bound to a particular region, the higher ATAC-seq, GRO-seq and H3K27ac signals can be detected (Figure 1F).

Taken together, these results show that (i) chromatin openness can be accurately predicted, without any prior biological knowledge, from occupancy values using all five TFs, suggesting that the most important TFs were identified and included in the model; (ii) in the ‘full model’ the relative importance of PU.1 occupancy is one of the lowest among the studied TFs, which suggests that PU.1 binds pervasively low accessible regulatory regions as well.

### PU.1-labelled regulatory elements are widespread in macrophages exhibiting low transcriptional activity and histone acetylation

**Figure 2.**
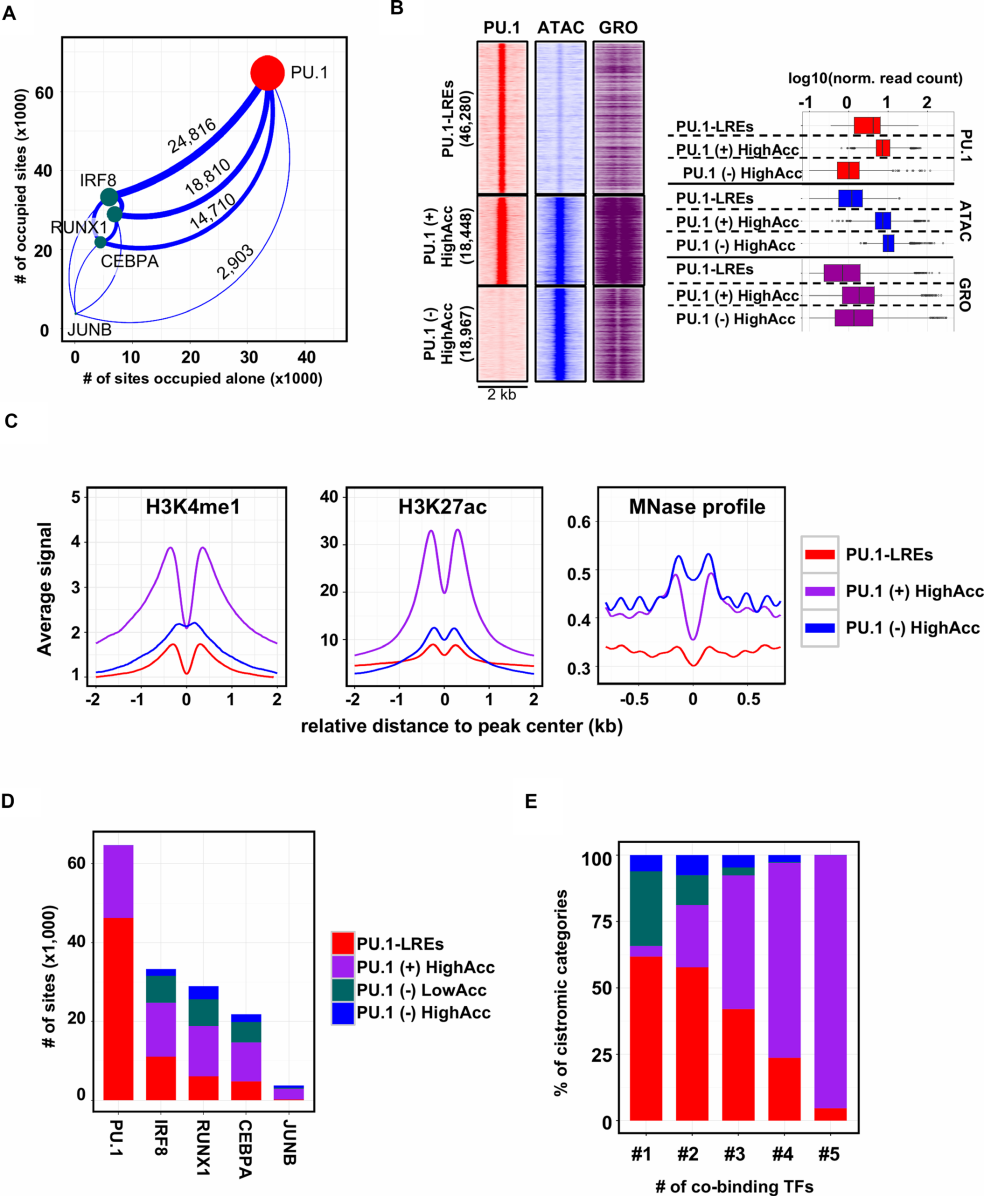
PU.1-labelled regulatory elements are pervasive in macrophage, exhibiting low transcriptional activity and histone acetylation. (**A**) Scatter plot showing the total number of binding regions for each transcription factor (TF) cistromes (*y*-axis), and the number of sites where the particular TF does not overlap with any other TF (*x*-axis). The overlap of the PU.1 cistrome with all the presented TF cistromes is represented by the thickness of the connecting lines on which the actual numbers of overlapping genomic regions are shown. The sizes of the dots are proportional to the sizes of the TFs’ cistromes. (**B**) Read distribution (RD) plots showing PU.1 occupancies, chromatin openness (ATAC-seq) and nascent RNA transcription (GRO-seq) in the three groups clustered based on PU.1 occupancy and ATAC-seq signal. The following nomenclature was used for the three clusters: PU.1-labelled regulatory elements (PU.1-LREs), PU.1 positive highly accessible regions (PU.1 (+) HighAcc) and PU.1 negative highly accessible regions (PU.1 (−) HighAcc). RD plots show the signals in 2-kb windows around the summit of PU.1 or ATAC peaks. Box plot representations of PU.1 occupancy and chromatin accessibility in the three clusters from ChIP-seq and ATAC-seq experiments are also shown. (**C**) Meta histograms showing H3K4me1, H3K27ac signals and MNase-seq (38) signals for the three categories. Signals were measured around the summits of PU.1 (PU.1-LREs and PU.1 (+) HighAcc) or ATAC-seq signals (PU.1 (−) HighAcc). (**D**) Stacked bar plots with the number of binding regions from each cistrome using PU.1, RUNX1, CEBPA, IRF8 and JUNB ChIP-seq experiments in the context of the identified categories (PU.1-labelled regulatory elements (PU.1-LREs), ‘PU.1 (+) HighAcc’, ‘PU.1 (−) LowAcc’ and ‘PU.1 (−) HighAcc’). (**E**) Stacked bar plot showing the percentage of binding sites in each cistromic category presented on panel (D) as a function of the number of co-binding factors present at the given genomic loci (#1-5 labelling indicates the number of co-bound TFs for each cistrome).

Next, we assessed the nature of interrelationship between the cistromes of the investigated TFs, and their capacity to bind alone at low accessible sites. Our analyses revealed that PU.1 had the largest cistrome (64 728 sites), but also had the highest fraction of binding sites that did not overlap with any other TFs (51.8%). In contrast, lower fractions of IRF8, RUNX1 and CEBPA (17.9%, 23.8% and 20.3%, respectively) had solo binding. Notably, only 4.5% of the total JUNB cistrome was able to bind alone, indicating that this TF is highly collaborative in the macrophage genome. IRF8 showed the highest overlap with PU.1 (24 816 sites), RUNX1 and CEBPA possessed an extensively shared cistrome with PU.1 (18 810 and 14 710 sites, respectively), and PU.1 occupied the vast majority (78%) of the JUNB cistrome (2903) (Figure 2A).

This analysis prompted us to classify the identified PU.1-bound genomic regions into three major categories: (i) binding sites that do not overlap with predicted open chromatin regions, termed ‘PU.1-labelled’ regulatory elements (PU.1-LREs, 46,280 sites); (ii) regions where PU.1 binding and chromatin openness overlap, termed ‘PU.1 pos. HighAcc’ sites (18 448 sites) and (iii) PU.1-negative highly accessible regions, termed ‘PU.1 neg. HighAcc’ sites (18 967 sites). Strikingly, only one-third of the PU.1-bound genomic regions were associated with open chromatin regions (Figure 2B and Supplementary Table S2). Comparison of the three categories with regard to enhancer RNA signal (Figure 2B), the general enhancer mark H3K4me1 and the active enhancer mark H3K27ac (Figure 2C) revealed that ‘PU.1 pos. HighAcc’ sites were greatly enriched for these features, while ‘PU.1 neg. HighAcc’ sites exhibited much lower enrichment for these features, and PU.1-LREs showed the lowest signals for these histone marks. These results suggest that these categories are indeed functionally distinct, and PU.1-LREs most likely represent a context where PU.1 needs additional collaborating TF(s) not present in the steady state to maintain or establish highly accessible chromatin. Next, we re-analysed a publicly available BMDM MNase-seq dataset (38), which revealed that the neighbouring nucleosomes of highly accessible sites (both PU.1 negative and PU.1 positive) showed higher MNase signal compared to the PU.1-LREs (Figure 2C). This suggests that nucleosomes are less positioned around PU.1-LREs, confirming low accessible chromatin conformation.

Pairwise comparison of the cistromes revealed that more than 50% of the TFs’ cistromes overlapped with the PU.1 cistrome, and the majority of these sites were associated with ‘PU.1 pos. HighAcc’ sites (Figure 2D). Notably, we identified a remarkable fraction of sites for IRF8, RUNX1 and CEBPA that were neither bound by PU.1 nor associated with highly accessible regions (‘PU.1 neg. LowAcc’), indicating the presence of LREs bound by these other TFs. This classification also showed a good correlation with our previously characterized co-binding events. The fraction of the ‘PU.1 pos. HighAcc’ sites gradually increased, while the fraction of PU.1-LREs and PU.1 neg. LowAcc (labelled by other TFs) regions gradually decreased with the number of co-bound TFs (Figures 1F and 2E). Finally, as an alternative approach to uncover the pervasiveness of LREs, we used ChromHMM, a Hidden Markov Model-based method (39). The analysis of the studied TFs coupled with ATAC-seq and ChIP-seq data for elongation-specific polymerase II (RNAPII-pS2), the active promoter mark H3K4me3 and another active regulatory element mark H4ac confirmed our observation: besides the active enhancer regions, various types of LREs were predicted, including PU.1-LREs, PU.1+IRF8 co-LREs and RUNX1-LREs (Supplementary Figure S2A).

Collectively, these results demonstrate that a large fraction of PU.1-bound regions are characterized by low accessible chromatin and the lack of transcriptional activity, which we termed LREs. Moreover, both the identification of ‘PU.1 neg. LowAcc’ sites and the ChromHMM-based analysis indicate that IRF8, RUNX1 and CEBPA also have labelled fractions and there might be unique combinations of these co-LREs.

### The cistromes of the key transcriptional regulators of the macrophage epigenome all have labelled regulatory elements

**Figure 3.**
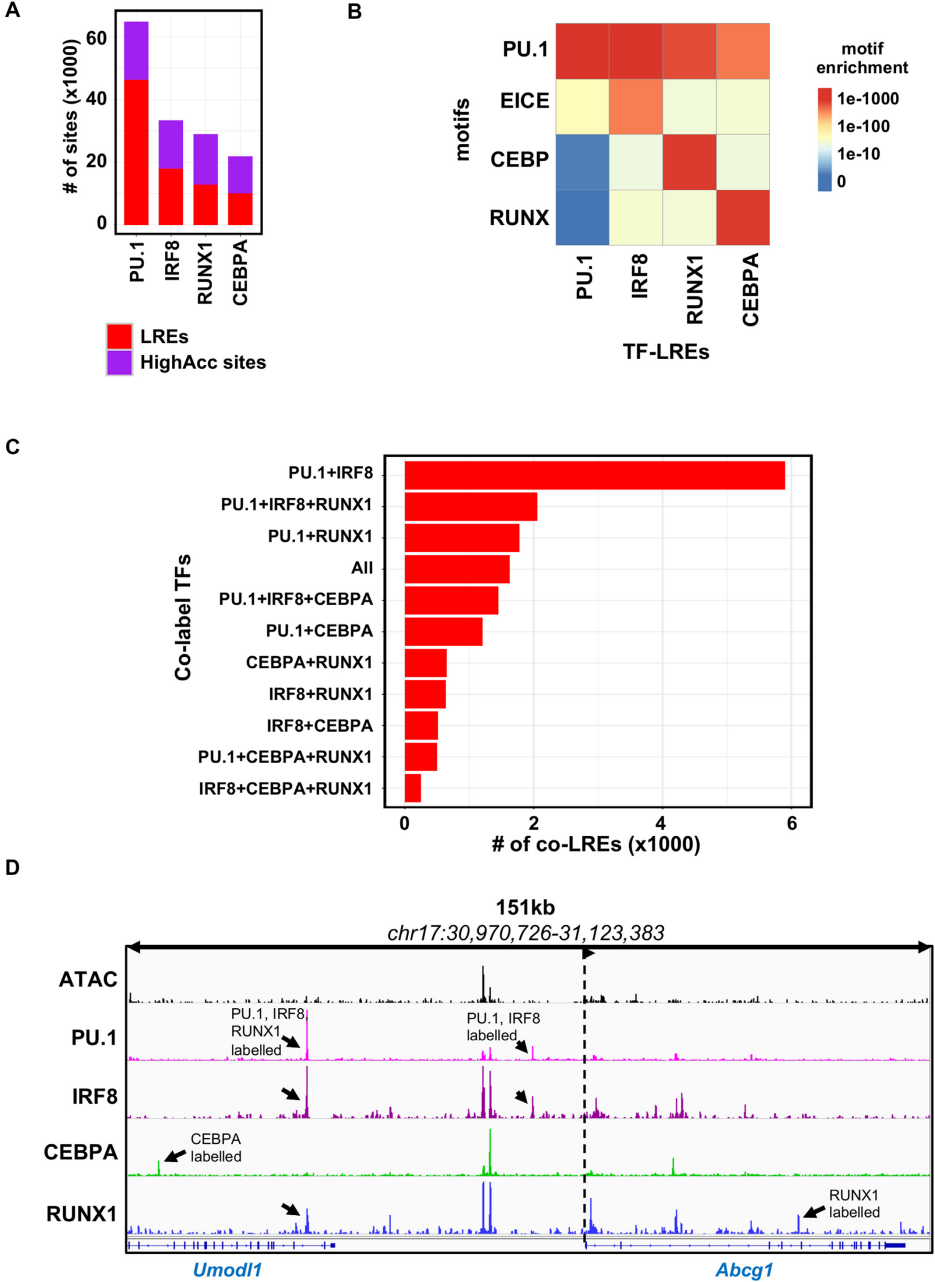
The principal transcriptional regulators of the macrophage epigenome form co-labelled regulatory elements. (**A**) Stacked bar plot showing the distribution of the transcription factor (TF) cistromes (PU.1, IRF8, RUNX1, CEBPA) on labelled regulatory elements (LREs) and highly accessible chromatin regions (HighAcc sites). (**B**) Heat map showing the *P*-values of motif (PU.1, EICE, CEBP and RUNX) enrichments of the redundant set of the different TF-LREs (PU.1, IRF8, RUNX1 and CEBPA). (**C**) Bar plot showing the number of different subsets of co-LREs by the TFs studied. (**D**) IGV snapshot of *Abcg1* locus with representative examples for different type of TF co-LREs. Black arrows highlight the different LREs and dashed lines with an arrow head represent the transcription start site and the direction of the gene.

To address the question whether having such a high fraction of LREs is specific to the PU.1 cistrome or it is a general phenomenon of the key TFs of the macrophage, we assessed how distal binding sites of IRF8, CEBPA and RUNX1 correlate with accessible chromatin. Due to its relatively small cistrome in the steady state, which has been also reported by others (40), and the low fraction of LREs, we decided to exclude JUNB from further analyses and concluded that probably JUNB represents a distinct class and does not label low accessible chromatin extensively. The comparison of the three remaining TFs revealed that, although these had smaller cistromes compared to PU.1, nearly half of their binding sites can be assigned to low accessible chromatin. These sites qualified to be classified as LREs (Figure 3A). Next, we compared the *P*-values associated with motif enrichments among the different types of LREs (Figure 3B). As expected, the specific motif of the TFs showed very high *P*-value in the corresponding TF-LRE group in each case. Interestingly, all the TF-LRE groups showed high enrichments for the PU.1 motif, underlining the unique genome organizing role of PU.1 in macrophages. This result also explains why the labelled fraction of IRF8, CEBPA and RUNX1 cistromes co-localize with labelled PU.1 regulatory regions (Figure 2D), while these three TFs showed moderate enrichments for the motifs of the other TFs.

In contrast, PU.1-LREs were less enriched for the motifs of the other three TFs, which can be explained by the higher number of the PU.1 binding sites at the genome-wide level. Notably, PU.1-LREs showed high enrichment for the EICE motif raising the possibility for a strong collaboration between PU.1 and IRF8 on LREs. To reveal the collaborative nature of the studied TFs, we analysed the co-binding of TFs at different types of LREs. We found that PU.1+IRF8 co-LREs were the most frequent (5901 sites) among all possible combinations. Moreover, in the top six most highly abundant combinations contained either PU.1 or IRF8, or both. In line with the finding that the highly accessible sites were correlated with a high number of co-bound TFs (Figure 2E), we detected a relatively low number of co-LREs that were bound by three or all four TFs (Figure 3C and D).

These results suggest that the existence of LREs is a general phenomenon for all the studied TFs, and PU.1-labelled sites are the most dominant LREs due to the larger cistrome of PU.1 and its higher capacity of binding alone. Thus, the labelled fraction of IRF8, CEBPA and RUNX1 often co-localize with PU.1, establishing co-LREs. Finally, among the possible combinations of co-LREs, PU.1+IRF8 co-LREs are the most abundant, having around three times as many binding sites than the second most abundant combination, in which PU.1 and IRF8 also participate.

### PU.1 and IRF8 transcription factors are important for the cellular response to IL-4 and form co-labelled regulatory elements at gene loci associated with alternative polarization marker genes

After making the surprising observation that there is a high number of PU.1+IRF8 co-LREs in the resting macrophage genome, we were wondering about the possible roles of these regulatory elements in IL-4-mediated macrophage polarization (alternative macrophage polarization). Signal Transducer and Activator of Transcription 6 (STAT6) almost exclusively drives the early steps of the polarization process (41) extensively populating the genome within minutes, triggering a robust gene expression signature; therefore, we stimulated the macrophages for 1 h with IL-4. We hypothesized that there may be PU.1+IRF8 co-LREs that may have directing roles in the execution of signal-dependent genomic programs at some of the marker genes during the polarization process.

**Figure 4.**
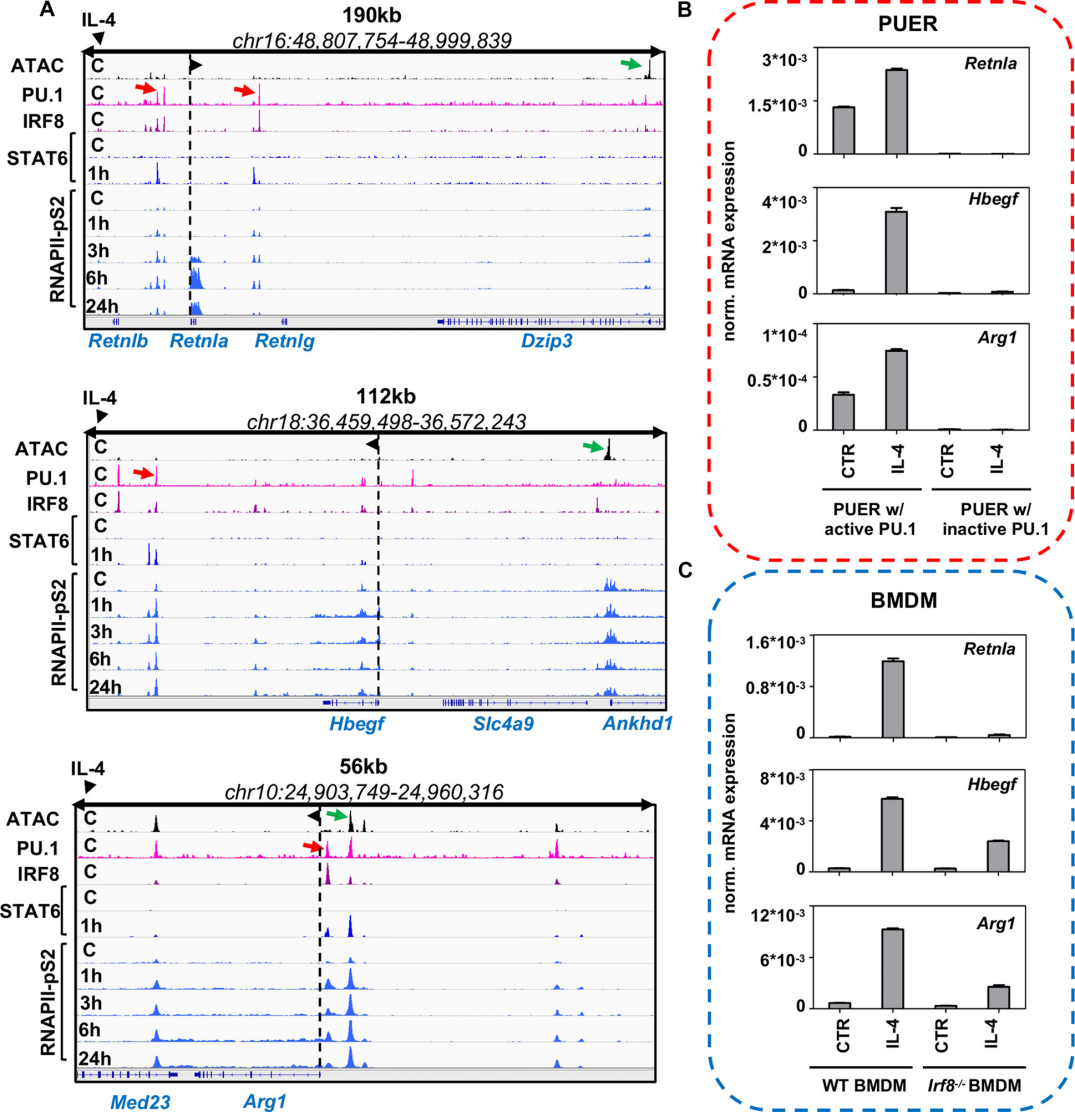
PU.1+IRF8 co-labelled regulatory elements are important for the cellular response to IL-4. (**A**) IGV snapshots of three alternative macrophage polarization specific marker gene loci (*Retnla, Hbegf* and *Arg1*) with PU.1+IRF8 co-labelled regulatory elements (PU.1+IRF8 co-LREs) that bind STAT6 upon IL-4 treatment and recruit the elongation-specific form of RNA polymerase II (RNAPII-pS2). Green arrows represent highly accessible chromatin regions, while red arrows depict PU.1+IRF8 co-LREs. Dashed lines with arrowheads represent the transcription start site and the direction of the gene. (**B**) Gene expression measurements by RT-qPCR at the mRNA level on the three alternative polarization marker genes (*Rentla, Hbegf* and *Arg1*) from PUER cells. PUER cells were exposed to tamoxifen for 24 h (PUER w/ active PU.1) or left untreated (PUER w/ inactive PU.1) followed by 3 h of IL-4 stimulation. The expression of mRNAs are normalized to the expression of the housekeeping gene *Ppia*. (**C**) Gene expression measurements of alternative polarization marker genes (*Rentla, Hbegf* and *Arg1*) by RT-qPCR at the mRNA level from wild-type bone marrow-derived macrophages (WT BMDM) or *Irf8*-deficient BMDMs (Irf8^−/−^ BMDM) after exposed to IL-4 for 3 h. Expression values are normalized to *Ppia*.

Having mapped the activated distal enhancer regions associated with LREs using fine resolution, time-course RNAPII-pS2 ChIP-seq experiments in IL-4-treated BMDMs (1, 3, 6 and 24 h), we observed that in the vicinity of important IL-4 regulated genes such as *Retnla, Hbegf* and *Arg1*, PU.1+IRF8 co-LREs can often be found and activated by STAT6 (Figure 4A). These results show that PU.1+IRF8 co-LREs can be transformed into active enhancers upon alternative polarization by IL-4, and raises the question whether both PU.1 and IRF8 are necessary to be present at these sites to properly mediate the gene expression program of STAT6.

Using two different systems (gain and loss of function), we embarked on providing a detailed picture of the possible role of PU.1+IRF8 co-LREs in gene regulation. We measured three IL-4 regulated genes (*Retnla, Hbegf* and *Arg1*) at the mRNA level in Pu.1^−/−^ myeloid progenitor cells containing a PU.1-estrogen receptor ligand binding domain fusion protein (PUER cells). The transcriptional activity of this fusion protein can be switched on in the presence of tamoxifen. Our experiments in the PUER system revealed that active PU.1 significantly increased the expression of all studied genes at the steady state, and provided the context for efficient IL-4-mediated induction as well (Figure 4B). Moreover, to test the role of IRF8 in IL-4 regulated expression of the same three genes, we used wild-type (WT) and *Irf8*^−/−^ BMDMs (Figure 4C). Our result showed that the lack of *Irf8* caused partial (*Hbegf* and *Arg1*) or complete (*Retnla*) loss of inducibility by IL-4 treatment (Figure 4C).

These results show that PU.1+IRF8 co-labelled sites are present around a selected set of alternative polarization marker genes, and these sites are actively utilized upon gene activation. Both PU.1 and IRF8 had significant effects on IL-4-induced gene expression, suggesting the involvement of the PU.1+IRF8 co-LREs in gene regulation. Future studies utilizing genome engineering methods should provide direct evidence regarding the importance of these co-LREs.

### IRF8 maintains low accessible chromatin structure at a subset of labelled regulatory elements

Our previous results confirmed that a significant fraction of the IRF8-binding sites bind to low accessible chromatin. We have also shown that IRF8 is required for IL-4-mediated gene expression, but the way its loss affects chromatin openness has not been investigated. In order to address this question, we performed ATAC-seq experiments in resting *Irf8*^−/−^ macrophages. Global analysis on distal regulatory elements bound by IRF8 in WT BMDM identified 3323 regions with lower chromatin openness, while 2322 sites gained openness in the absence of *Irf8* (Supplementary Figure S3A and Supplementary Table S3).

Motif analysis on regions with decreased chromatin openness identified the typical macrophage-specific TF motifs (TRE, PU.1, RUNX and CEBP) along with a strong PU.1-IRF composite motif (EICE), while under the sites where openness was increased, we did not detect the PU.1-IRF composite motif or any other ISRE-like motifs. Instead, we detected enrichment for CEBP, ETV (ETS variant 3) and NF-kB motifs; however, the latter two with much weaker *P*-values.

Interestingly, 37% of sites gaining openness in the absence of *Irf8* were IRF8-LREs, indicating that binding of IRF8 is necessary to prevent chromatin opening at these sites in WT BMDM. This analysis suggests that the lack of IRF8 affects the chromatin structure, and IRF8 might have a chromatin compacting effect on a subset of loci, potentially via either indirect binding or binding to non-canonical motifs (Supplementary Figure S3B,C,D).

Our results confirm the importance of IRF8 as a regulator of chromatin structure and point to the existence of PU.1+IRF8-co-LREs and IRF8-LREs, where IRF8 may maintain low accessible chromatin structure, thus stabilizes the LRE state. Whether and how signal-dependent transcriptional programs work at these LREs in the absence of IRF8 require further investigation in future studies.

### Labelled regulatory elements are dynamically utilized by the transcriptional programs of signal-dependent macrophage polarization

Finally, we investigated whether and how LREs are utilized upon classical and alternative macrophage polarization. For this purpose, we compared the cistromes of the previously studied SDTFs STAT6 and p65, the two main initiators of the alternative and classical polarization programs, respectively. The p65 protein is a member of the NF-κB TF complex, which turns on the pro-inflammatory program of macrophages following bacterial (LPS, lipopolysaccharide) stimulation.

**Figure 5.**
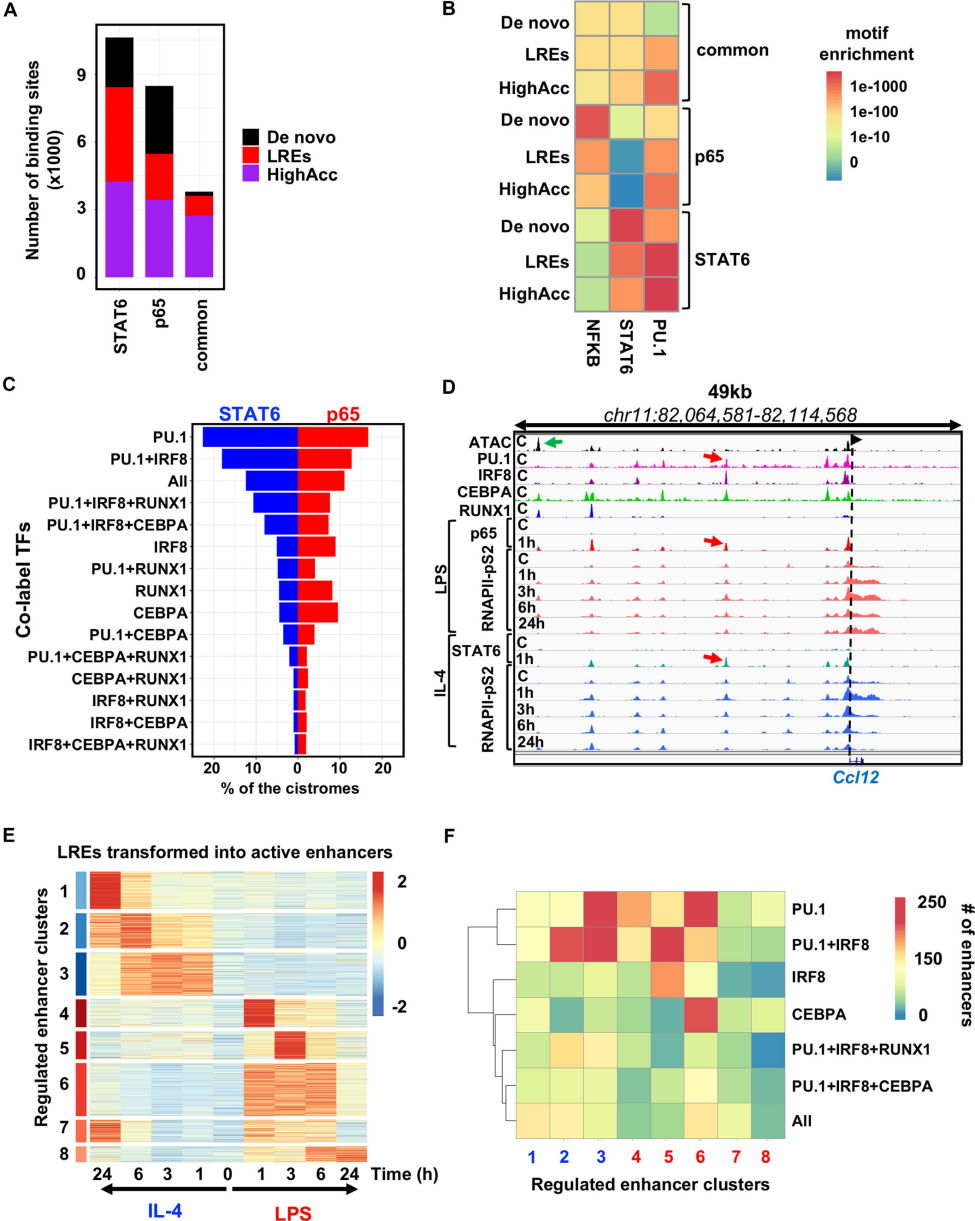
Labelled regulatory elements are extensively and dynamically utilized by the signal-dependent transcriptional programs of macrophage polarization. **(A**) Stacked bar plot showing the distribution of *de novo*, labelled regulatory elements (LREs) and highly accessible chromatin regions (HighAcc) across STAT6-specific, p65-specific and commonly bound genomic regions by the two signal-dependent transcription factors (SDTFs). (**B**) Heat map showing the *P*-values of the motif (NFKB, STAT6 and PU.1) enrichments for the sets of *de novo*, LREs and HighAcc chromatin regions. (**C**) Balance bar plot showing the distribution of the STAT6 and p65 cistromes on the different subsets of transcription factor co-labelled regulatory element groups. TF combinations that co-label these LREs are presented on the *y*-axis, while the *x*-axis depicts the percentages of the STAT6 and p65 cistromes that overlap with them. (**D**) IGV snapshot of *Ccl12* locus with a TF co-labelled regulatory element that can be used by both SDTFs (STAT6 and p65) and each of these two recruit the elongation-specific form of RNA polymerase II (RNAPII-pS2) with different kinetics upon LPS and IL-4 mediated macrophage polarization. (**E**) Heat map representation of the transformation of labelled regulatory elements (LREs) to active enhancers based on RNAPII-pS2 recruitment upon LPS or IL-4 mediated macrophage polarization on the time course of 1, 3, 6 and 24 h. Regulated enhancer clusters (ECs) (FC > 2 & *P*-value < 0.05) and normalized *Z*-scores of RNAPII-pS2 signals are shown. (**F**) Heat map depicting the highly enriched TF-LREs in the polarization induced enhancer clusters determined on panel (E). TF-LREs at least with 100 overlapping sites are shown.

Comparison of the cistromes of STAT6 (1 h IL-4) and p65 (1 h LPS) (Supplemental Figure S4A) revealed 10 619 STAT6-specific, 8466 p65-specific regions and 3781 commonly bound genomic regions that are used by both TFs in the presence of their activating signals. Both TF cistromes had similar fractions of *de novo*, LRE- and highly accessible chromatin-bound fractions (Figure 5A). Common genomic regions were enriched mainly for highly accessible sites (2720) but a smaller set of labelled (892) and a very low number of *de novo* sites (169) were also detected (Figure 5A). Motif strength analysis of NF-κB (represented by p65), STAT6 and PU.1 showed that (Figure 5B): (i) the PU.1 motif is weaker in almost all the cases on *de novo* sites and LREs compared to highly accessible sites, regardless of whether it is bound by STAT6, p65 or both; (ii) the NF-κB motif is strong at p65-specific sites and among these, *de novo* regions possess the strongest motifs. In addition, common sites show a much weaker NF-κB motif, while the motif can be rarely detected at STAT6-specific sites; (iii) the STAT6 motif exhibits very similar characteristics to the p65 motif among the groups, showing specificity to STAT6-bound regions with the strongest motifs detected under *de novo* sites. LREs have similarly strong motifs to *de novo*, while highly accessible chromatin regions harbour the weakest motifs. (iv) Commonly bound regions by STAT6 and p65 show the least specific motifs for the two factors, while 72% of these sites are highly accessible, 24% are LREs and only 4% are *de novo*.

These results suggest that the ability of SDTFs to bind and open chromatin is not entirely sequence-specific and the chromatin context such as highly accessible regions and LREs may also allow for additional binding and chromatin remodelling options where the motif is weaker.

Next, we turned our attention to analysing how the cistromes of STAT6 and p65 utilize LREs. To address this question, we mapped the cistromes of the two SDTFs to the identified transcription factor-LREs (TF-LREs) (Figure 5C). Both the STAT6 and p65 cistromes co-localized extensively with PU.1-, PU.1+IRF8- and PU.1+IRF8+CEPBA+RUNX1-LREs (‘All’ group); however, we found that a higher fraction of STAT6 utilized these groups than p65. Notably, p65 were more enriched for IRF8-, RUNX1- and CEBPA-LREs than STAT6, indicating that in certain cases, SDTFs preferentially use one or more LRE groups (irrespectively of the number of the co-bound TFs). Taken together, these analyses confirm that LREs can be bound by the SDTFs of the two main polarization programs and suggest a so far underappreciated scenario, where the most collaboratively occupied LREs are not necessarily favoured by the SDTFs, and show preference to other, more restricted TF constellations at LREs.

Our results imply that LREs play an important role in mediating short-term polarization signals. To find out whether these groups have any contribution to intermediate or long-term transcriptional changes as well, we mapped the dynamic, active enhancer network of classically and alternatively polarized macrophages using a fine resolution, time-course ChIP-seq experiment for RNAPII-pS2. We exposed macrophages to IL-4 and LPS for 1, 3, 6 and 24 h, and as exemplified by the *Ccl12* locus, an LRE is dynamically activated by both polarization programs (Figure 5D). We detected 6356 LREs that were up-regulated upon IL-4 or LPS stimulus at least at one time point compared to the steady state (FC > 2 and *P*-value < 0.05, Supplementary Table S4). Cluster analysis of the up-regulated LREs resulted in eight enhancer clusters (ECs), all of which had different transcriptional kinetics: EC1–EC3 were specific for IL-4 while clusters EC4–EC8 were LPS-specific (Figure 5E). Based on the distribution of cluster sizes, LREs were found to nearly equally contribute to early, intermediate and late transcriptional responses.

These results raised the question of whether there are specific TF motif co-occurrences, associated with certain kinetics. As expected, motif analysis of the ECs revealed that the PU.1 motif was enriched in all clusters, while the STAT6 motif was enriched in EC2 and EC3, the early and intermediate responsive clusters of the IL-4 signal, and NF-κB for EC4 and EC6, which were rapidly induced by LPS at 1 h. We identified motifs associated with the EGR family of TFs in EC1 (late responsive IL-4 clusters) and the Microphthalmia-associated (MAF) TF family in EC2 and EC3 (early and intermediate responsive IL-4 clusters), as proposed by recent studies (42,43), as well as MITF and RUNX under the IL-4 specific ECs. In contrast, TRE and CRE motifs bound by certain members of the AP-1 family showed higher enrichments in the LPS intermediate (EC6 and EC7) clusters while the motif of CEBP was enriched in the late responsive group (EC8) as well. ISRE and EICE bound by the members of interferon-regulatory factors (IRFs) in homo- or heterodimeric forms or together with PU.1, respectively, were over-represented both in IL-4 specific early and intermediate ECs (EC2 and EC3) and in EC5, one of the intermediate response set of LPS-induced LREs (Supplementary Figure S4B).

To confirm that these labelled ECs regulate gene expression with similar kinetics, we selected the genes based on two criteria: (i) on the gene body of the gene, Pol II S2 signal was up-regulated upon IL-4 or LPS at least at one time point compared to the steady state (1735, FC > 2 & *P*-value < 0.05) and 2), their TSSs could be associated with at least one regulated EC in a 100-kb-wide window. The associated genes clustered into eight gene body clusters (GBs), and the associated sets showed similar expression kinetics (Supplementary Figure S4C and D). Gene Ontology analysis of GBs revealed that both IL-4-specific GBs were enriched for immune system modulation-related terms, such as ‘regulation of cell adhesion’ or ‘leukocyte migration’, general activation-related terms such as ‘response to external stimulus’ and selectively enriched for alternative activation-related terms such as ‘CD4-positive alpha-beta T cell activation’. In contrast, LPS-specific GBs were enriched for classical activation-related terms such as ’regulation to interferon beta’ and ‘defense response’ (Supplementary Figure S4E). The binding patterns of the highly enriched LREs (at least with 100 overlapping sites with any ECs) was in agreement with the motif enrichments: PU.1+IRF8+RUNX1 tended to have more overlapping regions with IL-4 specific clusters and PU.1+IRF8+CEBPA was shifted towards an LPS-specific cluster (EC5), while ‘All’ LREs showed moderate enrichments for both IL-4 and LPS ECs. In contrast, PU.1 and PU.1+IRF8 LREs binding showed high and specific enrichments in both IL-4 and LPS early and intermediate groups (EC2,3 and EC4,5,6, respectively). Surprisingly, IRF8–LREs was strongly specific for the LPS-specific early responsive group, EC5. Similarly, CEBPA–LREs were highly enriched in the LPS-specific intermediate group (EC6).

Overall, our results indicate that LREs are dynamically utilized upon SDTF binding and activation both in classically and alternatively polarized macrophages, which show preference towards specific TF-LREs. In conclusion, LREs are pervasive features of the genome, representing a novel state of the potential enhancer repertoire of macrophages and contributing to dynamic gene expression regulation.

## DISCUSSION

Data integration, interpretation, conceptualization, and, ultimately, modelling have become a great challenge with the emergence of next-generation sequencing (44). Since capturing the complexity of the real world is impossible, building meaningful models with predictive values are essential to understand the mechanisms of a given biological process. Constructing algorithms that can recognize patterns in vast datasets provide unique ways to verify hypotheses (45,46). Here we utilized machine learning methods to make predictions on the relationship between TF binding (ChIP-seq for PU.1, IRF8, JUNB, CEBPA and RUNX1) and chromatin openness (ATAC-seq) in resting macrophages. Having learned patterns from the training set, machine learning methods made high-accuracy predictions, providing two important conclusions: (i) The TF panel we used in the machine learning approach predicted open chromatin openness with a very high (82%) accuracy, suggesting that these factors determine chromatin openness in macrophages and (ii) any of the TFs alone were not able to predict open chromatin with high efficiency, underlining the importance of collaboration between TFs and the existence of TF-bound, low accessible or closed chromatin, which we termed labelled regulatory elements (LREs). Integration of chromatin openness information and PU.1 binding identified PU.1-LREs, exhibiting low levels of histone methylation and acetylation. LREs are not only the specific feature of the PU.1 cistrome, but rather a widely occurring phenomenon of TF cistromes, although in our study the PU.1 cistrome has the largest fraction of LREs. In general, LREs exhibit strong motifs for the respective TF, which is most probably required for the given TF to be able to bind at these low accessible regions.

Most of the studies so far concerning collaborative binding suggested that some of the key, cell type-specific TFs must bind together to establish open chromatin and/or maintain this chromatin state. The discovery of LREs adds an extra layer to this concept by showing that the main TFs of the macrophage often bind chromatin in the absence of chromatin opening. In accordance with previous reports, our results also suggest collaborative binding as the main mechanism of chromatin structure remodelling and enhancer activation, but the discovery of LREs indicate the presence of a state, where collaborative TFs are present without opening chromatin. There is a large fraction of LREs, which are marked by only one of the studied TFs, but we also observed LREs where two, three or all four of the TFs are present. This observation suggests that the remodelling of chromatin structure does not necessarily happen even if collaboratively acting TFs are present. Importantly, the more TFs bind to a genomic region, the less likely it is to be associated with LREs (Figure 2E). This finding is compatible with the general model of collaborative binding, but points to the existence of a likely temporary chromatin state that seems to have been overlooked and underappreciated so far, characterized by TF binding to low accessible or closed chromatin. In our study, the myeloid LDTF PU.1 is the most prominent example of the LRE concept, because among the TFs investigated, PU.1 has the highest number of sites occupied alone. Importantly, the PU.1 cistrome labels regulatory elements in two-thirds of the cases. The cistromes of other TFs (IRF8, RUNX1 and CEBPA) contain LREs in approximately half of the cases, but on average two times fewer LREs can be associated with these cistromes than to the PU.1 cistrome. This underlines the dominance of PU.1-LREs in macrophages.

Studying the constellations of TFs on LREs identified PU.1+IRF8 co-LREs as the dominant co-label of low accessible chromatin. Worth noting, that a similar phenomenon was observed for the glucocorticoid receptor (GR), that increased chromatin accessibility and enabled transient access for binding of other TFs upon binding (47). Our findings confirmed this with the provision that sequence specificity is still an important determinant for SDTF binding even in these cases (Figure 5B). Our results highlight that there are preferred cooperative interactions between TFs on LREs similarly as it has been described in adipocytes on the example of PPARG and CEBPA (48) with the difference that it does not necessarily lead to an open chromatin structure. Second, co-LREs provide a landing track for SDTFs and seem to assist their loading, since the studied p65 and STAT6 SDTF cistromes extensively bind to these genomic regions. Although LREs are affected by the macrophage polarizing signals and are transformed into active enhancers, genome engineering needs to be applied to some of the LREs to directly characterize their functionality.

Another interesting aspect of the proposed LRE concept is whether labelling TFs cannot open chromatin at these genomic regions and provide epigenomic ‘find me signals’ for SDTFs or, at least on a subset of LREs, there is an active process of maintaining low accessible chromatin by these factors. Mapping of IRF8-related chromatin openness in Irf8^−/-^ macrophages suggests that thousands of IRF8–LREs and co-LREs exhibit more accessible chromatin structure in the absence of IRF8. This finding suggests that LREs enrich specific combinations of TFs to facilitate certain signalling events and they might have a role of outcompeting others from the TF pools present in the cells. Future studies are needed to design and execute experiments to provide molecular details on this phenomenon in relevant knockout models.

Recent studies have gone to quite a distance to characterize different states of the enhancers in the unstimulated macrophage and also upon activation (5,18). There are *de novo* or latent sites strongly dependent on the corresponding SDTF in a sequence-specific manner; the LDTFs can neither open nor bind these sites in the steady-state cell. Poised enhancers are already open prior to stimulation. Thus, although they are strongly bound by the available TFs in the unstimulated state, they require the SDTF(s) to become activated. The constitutively highly accessible sites are already active without external stimuli, and in some cases, the binding of the SDTF(s) can induce further activation. This class requires a lower level of sequence-specificity compared to the *de novo* or LREs, suggesting that open chromatin has a distinct role in recruiting SDTFs via less-specific DNA binding and/or protein–protein interactions.

Previously, the combination of PU.1 binding and H3K4me1/2 was used to define enhancer-like regulatory regions and active histone marks such as H3K27ac to discriminate between poised and active enhancers (5,49). This study utilizes ATAC-seq technique for directly profiling chromatin openness and extends the existing classification of activated enhancers by characterizing inducible PU.1-bound sites that are not associated with highly accessible chromatin in the steady state. Characterization of the LREs in the context of IL-4- and LPS-mediated polarization showed that a significant portion of these regulatory elements becomes active by the collaborative action of LREs and certain SDTFs such as STAT6 or p65 (Figure 5A).

Our recent work in embryonic stem cells also supports the notion of LREs. We demonstrated that OCT4 binds to a sizable set of low accessible regions and, at these sites, it is required for proper enhancer activation by recruiting co-regulators and RAR:RXR or β-catenin, similarly as co-LREs collaborate with STAT6 or p65 at low accessible sites upon external stimuli (50). Further refinement and extension of our understanding will be possible when genome-wide events can be studied at the single cell level (51,52) and in the timeframe of milliseconds instead of several minutes; hence we can avoid biases caused by population-based methods such as ChIP-seq (53,54).

In a broader sense, our conclusion is that there are two main pillars of SDTF binding. One is the epigenomic environment (highly accessible chromatin structure) formed by the key TFs of the cell type via collaborative binding without strong SDTF-specific motif sequences. Our new concept of LREs represents a novel layer of a permissive chromatin environment. The other is sequence specificity characterized by strong and specific motifs, which the SDTFs recognize, leading to the establishment of new regulatory regions that were neither highly accessible nor bound by the LDTFs before the stimulus (*de novo*). The LRE concept brings new insight in this regard too, because TF-LREs might have been considered *de novo* in previous studies, since the main TF (PU.1, IRF8, RUNX1 and CEBPA) cistromes of macrophages and chromatin openness have not been mapped at the same time.

Finally, analysis of the regulated enhancers in the context of two different polarization programs revealed that LREs are dynamically utilized in both classically and alternatively activated macrophages and regulate gene sets with similar transcriptional dynamics. Moreover, the two polarization programs studied show preference towards certain LREs, suggesting that the label or combinations of label TFs have specific, directing roles for SDTFs.

The need for computational tools to explain certain biological processes appeared in parallel with the emergence of experimental molecular biology, even before the Next Generation Sequencing Revolution. Therefore, molecular biology and computer science research communities need to collaborate and combine their tools; these interdisciplinary studies contribute to creating firmly established theories on complex biological processes and hold promise to identify hidden features of the genome like LREs.

## DATA AVAILABILITY

All data from this study have been submitted to Sequence Read Archive (SRA-NCBI; https://www.ncbi.nlm.nih.gov/bioproject) and to NCBI Gene Expression Omnibus (GEO; http://www.ncbi.nlm.nih.gov/geo/) under accession number PRJNA194083, PRJNA318630 and GSE106706, respectively. In-house scripts used to generate data and figures can be downloaded from the GitHub repository https://github.com/ahorvath/horvath_et_al_2019_labelled_enhancers.

## Supplementary Material

Supplementary Data
