# Supplementary material for: Labelled regulatory elements are pervasive features of the macrophage genome and are dynamically utilized by classical and alternative polarization signals
Source: Nucleic Acids Res. 2019 Feb 25;47(6):2778–92. doi: 10.1093/nar/gkz118 (PMC6451134; doi:10.1093/nar/gkz118)
Supplement: Supplementary Data [file gkz118_supplemental_files.zip › Horvath_et_al_NAR_Supplementary_Materials_final.pdf]

Supplementary Figure S1.

A

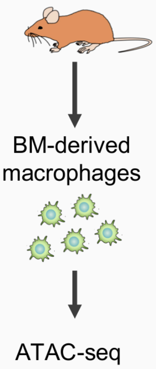

B

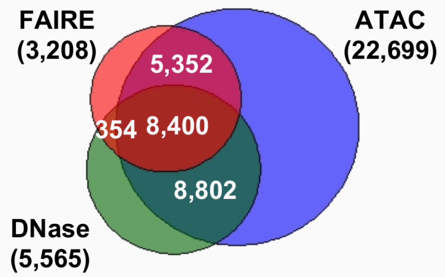

Experiment	Detected(Nr)	Detected(%)
**ATAC**	45,253/54,380	83.2%
**DNase**	23,121/54,380	42.5%
**FAIRE**	17,314/54,380	31.8%

C

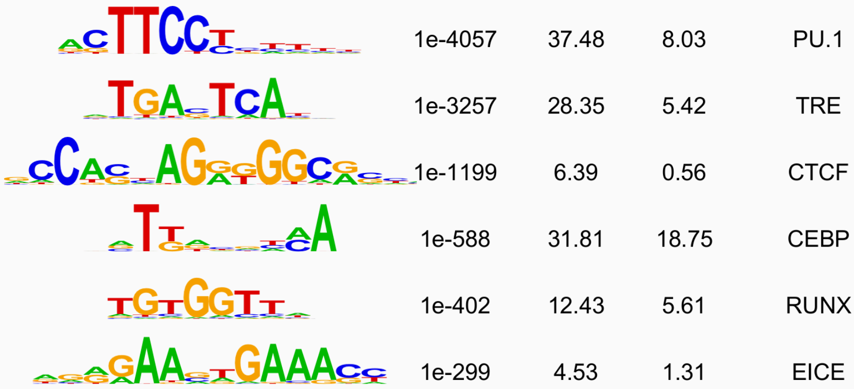

Motif logo	P-value	% of Target	% of Bg	Db. Annotation
	1e-4057	37.48	8.03	PU.1
	1e-3257	28.35	5.42	TRE
	1e-1199	6.39	0.56	CTCF
	1e-588	31.81	18.75	CEBP
	1e-402	12.43	5.61	RUNX
	1e-299	4.53	1.31	EICE

D

	Size	Sensitivity	Specificity	Accuracy
**Training Set**	2000	0.82	0.77	0.80
**Validation Set**	2000	0.88	0.77	0.82

Supplementary Figure S1.

(A) Experimental scheme of bone marrow-derived macrophage (BMDM) differentiation and identification of accessible chromatin regions by ATAC-seq. **(B)** Venn diagram showing overlap among the three techniques for profiling chromatin openness (ATAC-seq (this study), DNase-seq (1); and FAIRE-seq (2)). Table assessing the fraction of sites detected by a particular technique. Intersection was considered if at least one nucleotide overlapped. Proportional Venn

diagrams were generated with VennMaster. **(C)** The most enriched *de novo* motifs under the distal highly accessible chromatin regions. Motif sequence logos, P-value of enrichment, percentage of target sequences with motif (Targets (%)), and percentage of background sequences with motif (Bg. (%)) are shown. **(D)** Table showing the calculated measures of the Training and Validation sets used for Random Forest.

Supplementary Figure S2.

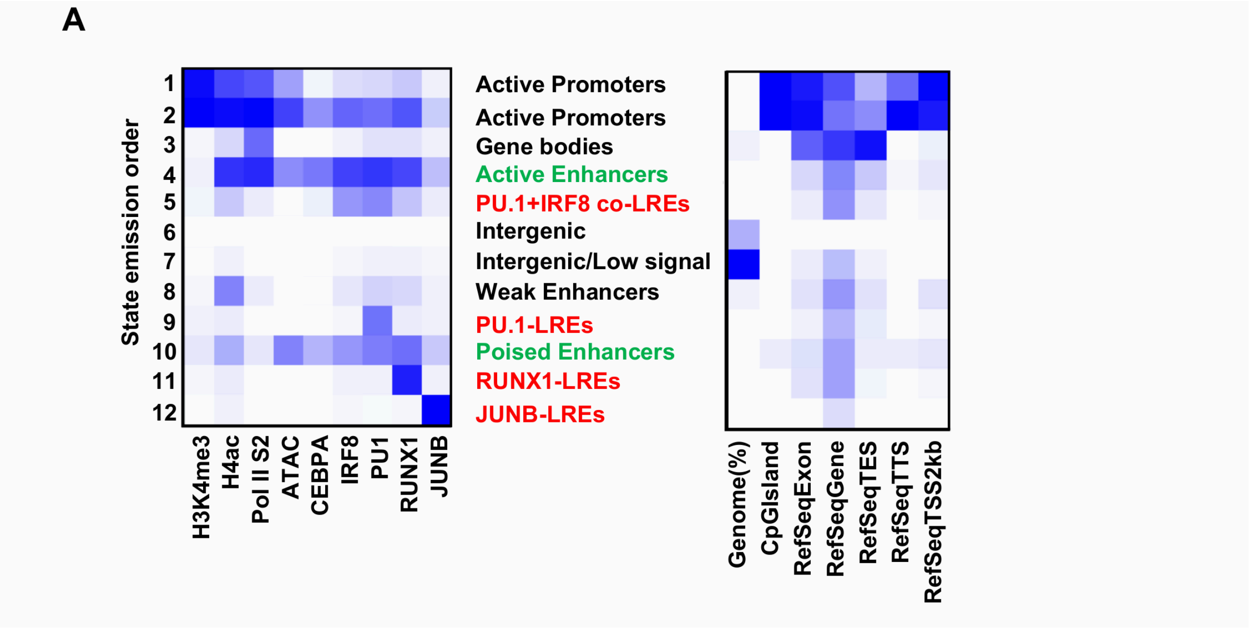

Supplementary Figure S2.

(A) Heatmaps showing the result of ChromHMM (3) analysis. 12 clusters were inferred from ATAC-seq, H3K4me3, H4ac, RNAPII-pS2, PU.1, IRF8, JUNB, RUNX1 and CEBPA ChIP-seq data as input variables (left panel) and the annotation of the inferred clusters according to RefSeq annotation (right panel).

Supplementary Figure S3.

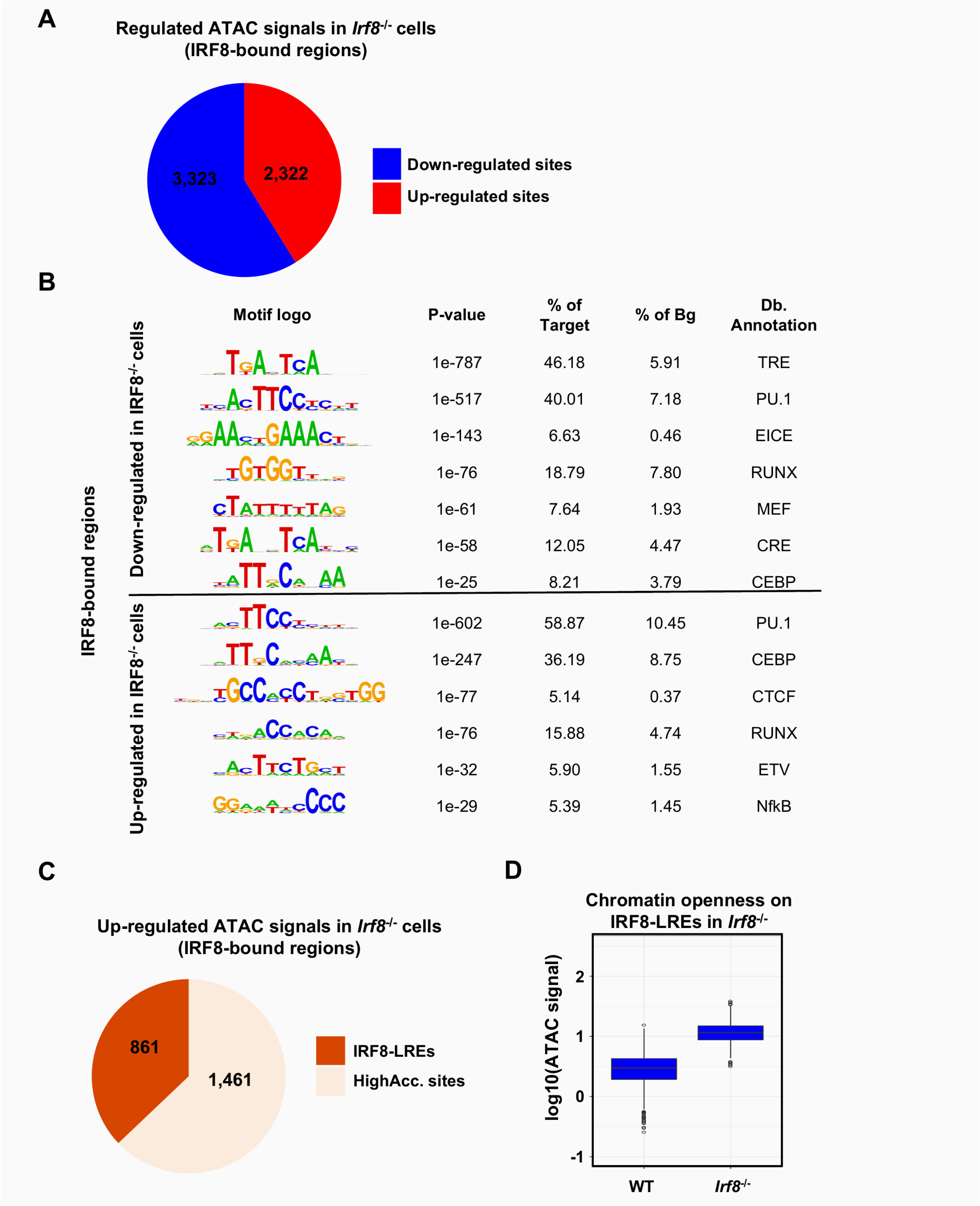

Supplementary Figure S3.

(A) Pie chart showing the number of IRF8-bound sites that were up- or down-regulated in *Irf8*$^{-/-}$ cells. DiffBind was used to infer differential binding sites (p-value < 0.05) from duplicates using WT samples as control. **(B)** The most enriched *de novo* motifs under IRF8-bound sites that were up- or down-regulated in *Irf8*$^{-/-}$ cells. Motif sequence logos, P-value of enrichment, percentage of target sequences with motif (Targets (%)), and percentage of background sequences with motif (Bg. (%)) are shown. **(C)** Pie chart showing the number of IRF8-LREs and HighAcc. sites among IRF8-bound regions gaining openness in *Irf8*$^{-/-}$ cells. DiffBind was used to infer differential binding sites (p-value < 0.05) from duplicates using WT samples as control. **(D)** Box plot showing chromatin accessibility of IRF8-LREs in WT and *Irf8*$^{-/-}$ cells.

Supplementary Figure S4.

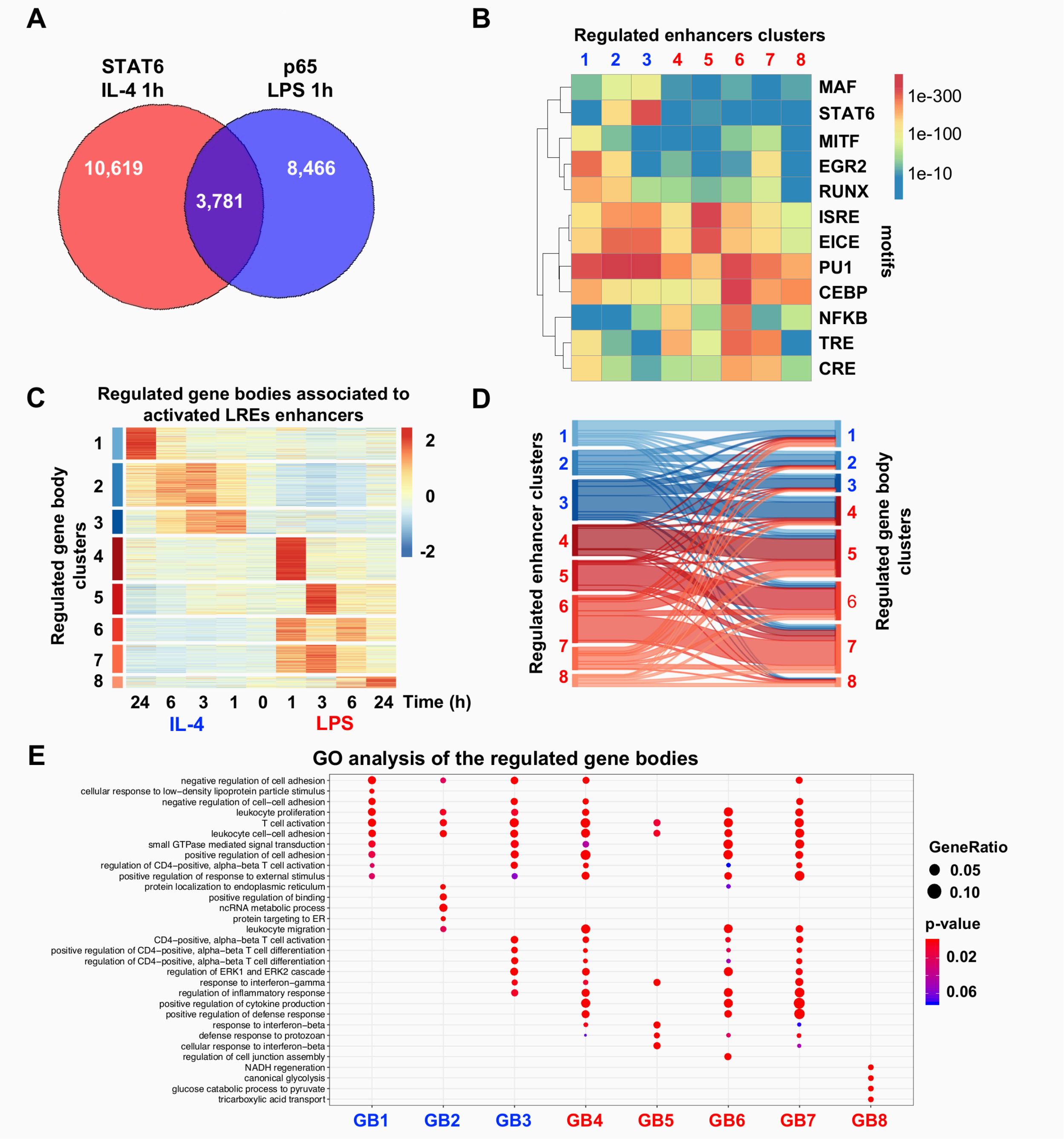

Supplementary Figure S4.

(A) Venn diagram showing the overlap between STAT6 and p65 cistromes induced by IL-4 or LPS, respectively. DiffBind analysis were performed separately for STAT6 and p65 experiments to infer differential binding sites (p-value < 0.05) from duplicates using untreated samples as control. **(B)** Heatmap showing *the* p-values of the motifs enriched under at least one of the identified ECs.

(C) Heatmap showing the differentially RNAPII-pS2 enriched gene bodies (GBs) following IL-4 or LPS stimulations over the time course presented. DiffBind was used to infer differentially regulated (FC>2 & p-value < 0.05) RNAPII-pS2 regions (1h, 3h, 6h, 24h IL4 and 1h, 3h, 6h, 24h LPS treatment) from duplicates using untreated samples as control. Normalized Z-scores of RNAPII-pS2 signals are shown. **(D)** Sankey plot showing the annotated gene-enhancer pairs from the 8 clusters defined for both the enhancers and gene bodies based on their different RNAPII-pS2 recruitment kinetics. The transcription start site of a gene body was associated to the regulated enhancer in a 200-kb-wide window. **(E)** Heatmap showing the enriched GO terms (Biological Processes) across GBs. The blue-red scale color contour represent p-values of the enrichments and the sizes of proportional to the ratio of the genes enriched for a certain term.

REFERENCES

1. Leddin,M., Perrod,C., Hoogenkamp,M., Ghani,S., Assi,S., Heinz,S., Wilson,N.K., Follows,G., Schonheit,J., Vockentanz,L., *et al.* (2011) Two distinct auto-regulatory loops operate at the PU.1 locus in B cells and myeloid cells. *Blood*, **117**, 2827–2838.
2. Ostuni,R., Piccolo,V., Barozzi,I., Polletti,S., Termanini,A., Bonifacio,S., Curina,A., Prosperini,E., Ghisletti,S. and Natoli,G. (2013) Latent enhancers activated by stimulation in differentiated cells. *Cell*, **152**, 157–171.
3. Ernst,J. and Kellis,M. (2012) ChromHMM: automating chromatin-state discovery and characterization. *Nat. Methods*, 10.1038/nmeth.1906.
